# Intrathyroidal parathyroid adenomas: Scoping review on clinical presentation, preoperative localization, and surgical treatment

**DOI:** 10.1002/hed.27287

**Published:** 2022-12-23

**Authors:** Shravan V. Gowrishankar, Rohan Bidaye, Tilak Das, Veronika Majcher, Brian Fish, Ruth Casey, Liam Masterson

**Affiliations:** ^1^ School of Clinical Medicine University of Cambridge Cambridge UK; ^2^ Department of Otolaryngology‐Head & Neck Surgery Cambridge University Hospitals NHS Foundation Trust Cambridge UK; ^3^ Department of Neuroradiology and Head & Neck Imaging Cambridge University Hospitals NHS Foundation Trust Cambridge UK; ^4^ Department of Endocrinology Cambridge University Hospitals NHS Foundation Trust Cambridge UK

**Keywords:** imaging techniques, intrathyroid parathyroid adenoma, lobectomy, thyroid and parathyroid surgery, ultrasound

## Abstract

Intrathyroidal parathyroid adenomas (IPAs) are a rare cause of primary hyperparathyroidism. They are often difficult to localize preoperatively and intraoperatively, making diagnosis and treatment challenging. Current data on IPAs are sparse and fragmented in the literature. This makes it difficult to compare the effectiveness of different imaging and surgical techniques. To address this issue, this scoping review maps the literature on IPAs, focusing on four domains: clinical presentation, current localization methods, different surgical techniques, and histopathological features. A search of MEDLINE, Embase, and the Cochrane Library was conducted, with 19 studies meeting the inclusion criteria. The characteristics of IPAs on ultrasound, fine‐needle aspiration, CT, MRI, sestamibi‐based techniques, and selective venous sampling are summarized. Emerging imaging modalities, including autofluorescence, are introduced. Surgical methods and intraoperative factors that correlate with high success rates for removal are highlighted. This review also identifies gaps in knowledge to guide further research into this area.

## 1 INTRODUCTION

Primary hyperparathyroidism (PHPT) is a common endocrine disorder caused by excess parathyroid hormone (PTH) production. The condition is caused by a solitary parathyroid adenoma up to 85% of the time, with parathyroid hyperplasia and carcinoma being less common causes.^1^ Typically, there are four separate parathyroid glands.^2^ Two glands are usually located posterior to the upper poles of the thyroid and are termed “superior” parathyroid glands; the remaining two glands have a more variable location but are typically located near the inferior thyroid poles and are termed “inferior” parathyroid glands. However, parathyroid glands can also be found at “ectopic” sites, owing to variations in migration during embryological development. In those with PHPT, the incidence of ectopic adenomas ranges from 9% to 22%, with a greater incidence where re‐operative cases are examined.^3^, ^4^, ^5^, ^6^ Common ectopic sites include the thymus, retropharyngeal space, retroesophageal space, and within the thyroid itself.^3^, ^7^

An intrathyroidal parathyroid adenoma (IPA) is an ectopic variant where the adenoma is either partly (>50%) or completely enveloped by the thyroid gland. The incidence of IPAs ranges from 0.7% to 6%.^1^, ^2^, ^3^ IPAs can be challenging to manage for a range of reasons. For instance, on imaging, they can appear similar to other structures in the thyroid, including benign thyroid nodules, making differentiation difficult. In the operating room, they can be challenging to find, being embedded in the thyroid. As a result, a thyroid lobectomy is often performed, sometimes with inconclusive prehistological evidence that an IPA is present and can lead to high failure rates.

There is a lack of consensus on which imaging techniques and surgical methods are best‐indicated in working up and managing this variant. One reason is that data on these factors are sparse and fragmented in the literature, which makes it difficult to compare techniques and draw recommendations for practice. To help address this issue, this scoping review aims to map the literature on IPAs, summarizing current imaging tools and highlighting unique imaging features. This review will also discuss the clinical presentation of IPAs, and important surgical considerations for intraoperative removal. It will also identify gaps in the literature to guide further research in managing this variant.

## 2 METHODS

The methodology was reported following the preferred reporting items for systematic reviews and meta‐analyses extension for Scoping Reviews (PRISMA‐ScR) checklist.^8^ A protocol for this study was not prospectively registered before commencing this review.

### 2.1 Data sources and search strategy

A literature search was performed on intrathyroidal parathyroid adenomas using PubMed through MEDLINE, Embase, and the Cochrane Library. A search for published and peer‐reviewed articles was performed using variants of the following keywords: “intrathyroid,” “intra‐thyroid,” “parathyroid,” “adenoma,” “parathyroid neoplasm,” and “primary hyperparathyroidism.” The full search strategy was developed with assistance from the University Medical Library team and is included in Data S1, Supporting Information. Due to the rarity of IPAs, the search strategy was designed to capture all articles on IPAs, which would then be individually reviewed to collect the categories of information outlined in Table 1.

**TABLE 1:** Articles were included if any of the following information could be extracted from intrathyroidal parathyroid adenomas (IPAs)

Categories of information collected	Details of information collected
Clinical presentation of IPAs	Symptoms on presentation
Localization techniques For example, Ultrasound Ultrasound‐guided fine needle aspiration Nuclear medicine techniques Magnetic resonance imaging Computed tomography Selective venous sampling	Accuracy of each technique in detecting IPAs Unique features of IPAs on imaging
Surgical considerations	Surgical techniques for removal Location of IPAs intraoperatively
Histopathology	Macroscopic features of IPAs Microscopic features of IPAs

Abbreviation: IPA, intrathyroid parathyroid adenoma.

### 2.2 Screening and data extraction

The search was run on the 30th of May 2022. Following de‐deduplication via Mendeley, all articles were initially screened by reviewing title and abstract. Relevant articles were then screened by reviewing the full text. These steps were performed by two authors independently (SG and RB), and disagreements were resolved through discussion. After full‐text screening, relevant articles were reviewed to extract the information and data items specified in Table 1. The data extraction form was designed prospectively on Microsoft Word and included (i) general study characteristics (author, year of publication, study type, sample size of IPAs), (ii) clinical features, (iii) localization techniques utilized, (iv) surgical considerations, and (v) histopathological features, as outlined in Table 1. Data from each eligible study was extracted by one author (SG) and was reviewed and cross‐checked by another author (RB). Disagreements were resolved through discussion.

### 2.3 Inclusion and exclusion criteria

Articles were included if information on clinical presentation, localization techniques, surgical considerations, and histopathology could be isolated from patients with IPAs. Further details on the categories of information collected is highlighted in Table 1. There were no restrictions made on patient age. Peer‐reviewed randomized control trials, cohort studies (with or without a comparison group), consecutive case series, and case–control studies were eligible for inclusion. Case reports, conference abstracts, literature reviews, and editorials were excluded. However, the references of excluded reviews were searched to identify articles which fit the inclusion and exclusion criteria (“snowballing”). Only English‐language studies were included. There were no limits set on the year published.

## 3 RESULTS

The results were reported following the preferred reporting items for systematic reviews and meta‐analyses extension for Scoping Reviews (PRISMA‐ScR) checklist.^8^

### 3.1 Study selection and characteristics of included studies

**FIGURE 1:**
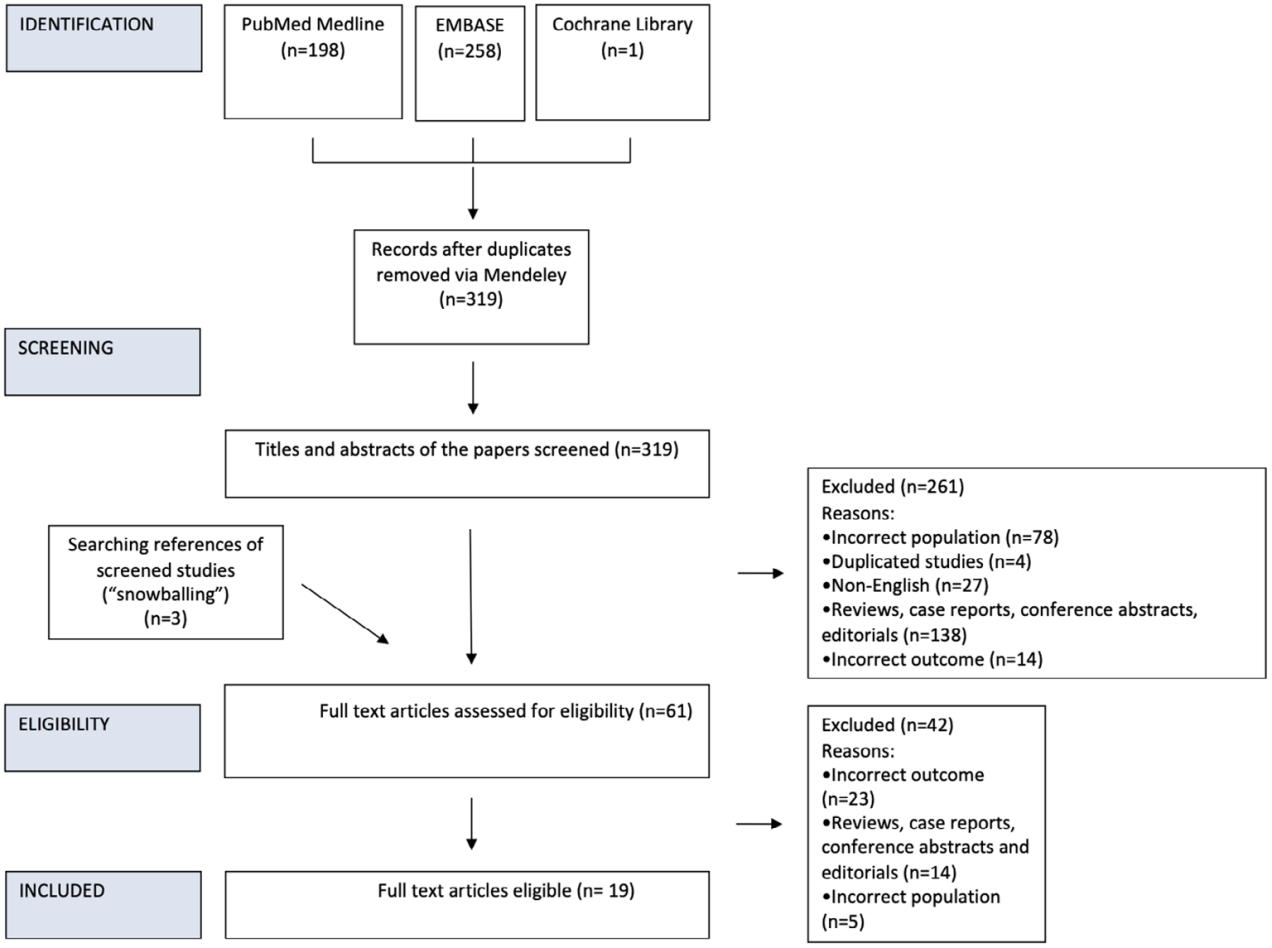
A flowchart outlining the screening process, including reasons for exclusion. This was reported based on the Preferred Reporting Items for Systematic Reviews and Meta‐Analyses Extension for Scoping Reviews (PRISMA‐ScR) [Color figure can be viewed at wileyonlinelibrary.com]

Electronic database searches resulted in 457 hits. Following de‐duplication, 319 articles were screened. Nineteen articles fit the inclusion criteria and were included in this review.^1^, ^3^, ^4^, ^7^, ^9^, ^10^, ^11^, ^12^, ^13^, ^14^, ^15^, ^16^, ^17^, ^18^, ^19^, ^20^, ^21^, ^22^, ^23^ Three of these articles were identified through reviewing the references of excluded literature reviews. A flowchart outlining the selection process, as outlined by PRISMA‐ScR,^8^ including reasons for exclusion, is shown in Figure 1. A summary of the characteristics of these 19 articles is provided in Table 2.

**TABLE 2:** Study characteristics of the 19 included studies

Study name	Year published	Study type	Number of IPAs	Differentiation between complete and partial IPAs	Domains of information included			
					Clinical features	Localization technique	Surgical considerations	Histopathology
Carral et al.^11^	2021	Retrospective cohort without control	3^a^	Not stated	–^b^	Yes, US‐guided fine‐needle aspiration	–	–
Ye et al.^12^	2018	Retrospective cohort without control	15	Differentiates between complete (12) and partial (3) IPAs	Yes	Yes, compares US, sestamibi‐SPECT, and CT	–	–
Chandramohan et al.^9^	2020	Retrospective cohort without control	10	Differentiates between complete (4) and partial (6) IPAs	–	Yes, accuracy of US	–	Yes, microscopic and macroscopic features outlined
Dream et al.^13^	2020	Retrospective cohort without control	43^c^	Not stated	–	Yes, accuracy of US, sestamibi and CT	Yes, outcomes after enucleation	–
Abboud et al.^14^	2007	Retrospective cohort without control	6	Includes complete (3) and partial (3) IPAs but analyses them together	–	Yes, accuracy of US	–	–
Herden et al.^15^	2011	Retrospective cohort without control	4	Not stated	–	–	Yes, intraoperative searching	–
Feliciano et al.	2007	Retrospective cohort without control	4	Includes complete (2) and partial (1) IPAs and 1 other was unspecified	–	–	Yes, discussion about partial vs. complete lobectomy vs. thyroidotomy	–
Yabuta et al.^17^	2011	Retrospective cohort without control	7	Includes complete and partial but does not specify numbers of each and analyses them together	–	Yes, unique features of US, accuracy of US, sestamibi, and CT	–	–
Roy et al.^7^	2013	Retrospective cohort without control	37	Not stated	–	Yes, accuracy of sestamibi and US	–	–
Zhao et al.^18^	2021	Retrospective cohort without control	13	Differentiates between complete (5) and partial (8) IPAs	–	Yes, accuracy of sestamibi SPECT–CT and US	–	–
Heller et al.^19^	2013	Retrospective cohort with control	50	Differentiates between complete (13) and partial (37) IPAs	–	Yes, unique features on US; prevalence of concurrent thyroid nodules; accuracy of US; accuracy of proposed criteria used to distinguish IPAs and thyroid nodules	–	–
Mazeh et al.^10^	2012	Case control study	53	Complete IPAs only	Yes	Yes, accuracy of sestamibi, US, CT, MRI, intraoperative PTH monitoring	Yes, information on intraoperative location	–
De La Cruz Vigo et al.^21^	1997	Retrospective cohort without control	6	Not mentioned	–	–	Yes, surgical recommendations on techniques	–
Thompson et al.^1^	1982	Retrospective cohort without control	5	Complete IPAs only	–	–	Yes, intraoperative location of IPAs	–
Goodman et al.^20^	2012	Retrospective cohort without control	192	Differentiates between complete (72) and partial (120) IPAs but only analyses complete IPAs	–	–	Yes, surgical outcomes for lobectomy and intraoperative location of IPAs	–
Shen et al.^22^	1996	Retrospective cohort without control	6	Not stated	–	Yes, accuracy of US, sestamibi, MRI, CT, selective venous sampling	–	–
Phitayakorn and McHenry^3^	2006	Retrospective cohort without control	6	Complete IPAs only	–	–	Yes, intraoperative location of IPAs	–
Mendoza et al.^4^	2010	Retrospective cohort with control	2	Not stated	Yes	–	–	–
Lebastchi et al.^23^	2015	Retrospective cohort without control	3	Not stated	–	Yes, selective venous sampling	–	–

Abbreviations: CT, computed tomography; IPA, intrathyroid parathyroid adenoma; MRI, magnetic resonance imaging; PTH, parathyroid hormone; SPECT, single photon emission computed tomography; US, ultrasound.

^a^ 3 IPAs were surgically confirmed.

^b^ No information provided.

^c^ 43 had single IPAs.

### 3.2 Clinical presentation

Patients with intrathyroidal parathyroid adenomas can present with overt symptoms of primary hyperparathyroidism, or can be asymptomatic.^9^, ^24^, ^25^ Symptoms of hypercalcemia are wide‐ranging and can include bone pain, polyuria, renal colic, constipation, and depression. In a cohort of 53 IPAs, Mazeh et al. found no difference in the type of clinical symptoms, demographics, and blood results relative to a control group of normal parathyroid adenomas.^10^ However, they did not note the severity of each symptom.

Due to difficulties in correctly diagnosing an IPA, patients can present with progressive symptoms of primary hyperparathyroidism over a prolonged period. Severe bone disease appears to be more prevalent with ectopic parathyroid adenomas, including IPAs.^4^ This might reflect delays in diagnosis and definitive management in patients with ectopic parathyroid adenomas.

Biochemical results usually show hypercalcemia and elevated PTH levels, although patients can be normocalcaemic. A palpable mass on neck examination is rare for parathyroid adenomas, including IPAs, and more indicative of thyroid pathology or parathyroid malignancy.^26^, ^27^, ^28^

### 3.3 Localization techniques

Twelve studies provided information on localization techniques (Table 2). Eleven studies provided information on ultrasound‐based techniques; 7 on sestamibi‐based imaging modalities, 5 on CT, 2 on MRI, and 2 on selective venous sampling.

#### 3.3.1 Ultrasound

The reported sensitivity of identifying intrathyroidal parathyroid adenomas using ultrasound (US) varies widely, with estimates ranging from 29% to 67%.^7^, ^9^, ^14^, ^19^ On ultrasound, IPAs can appear similar to other abnormalities such as benign thyroid nodules, which frequently co‐exist alongside IPAs.^19^ Features that point to an IPA include a solid lesion with an absence of cystic components, hypoechogenicity, and the presence of a single polar feeding artery on Doppler.^17^, ^18^, ^19^ Using these criteria in a cohort of 50 IPAs, Heller et al.^19^ reported that the sensitivity of identifying IPAs increased from 29% to 76% on a blinded review of the original imaging. In particular, a polar feeding vessel was an important differentiator, as this was present in 84% of IPAs, but in no thyroid nodules.

Another reported differentiating feature on US is a hyperechoic line on the ventral surface of the adenoma. Two case series with 6–15 IPAs found that around 85% of IPAs had a hyperechoic line,^12^, ^17^ which was not found in thyroid nodules.

On ultrasound, IPAs can also be seen as “complete IPAs,” where they are completely enveloped by thyroid tissue, or “partial IPAs” when >50% of the surface, but not the whole surface, is covered by thyroid tissue.^12^, ^19^ Partial IPAs appear to be more common than complete IPAs, and account for a larger proportion of IPAs missed by ultrasound.^9^, ^19^

However, ultrasound is operator‐dependent, and high success rates in detecting IPAs are associated with experienced radiologists.^9^, ^12^, ^19^

#### 3.3.2 Ultrasound‐assisted fine needle aspiration

Fine needle aspiration (FNA) of the lesion can provide information on cell type, theoretically aiding diagnosis. However, there is significant cytological and architectural overlap between parathyroid and thyroid tissue, making differentiation difficult on cytological analysis alone. Features previously thought to indicate thyroid tissue such as colloid, follicles, and perivacuolar granulation are present in a high number of parathyroid samples.^29^, ^30^ Many parathyroid adenomas also share features with follicular, papillary, and medullary thyroid cancer.^31^, ^32^

Positive immunocytochemical staining for chromogranin A (and synaptophysin) can be useful in delineating parathyroid cells,^33^ but a sufficient quantity of cellular aspirate is required. Chromogranin A is also present in other cell types derived from a neuroendocrine lineage—notably medullary thyroid cancer (MTC).^34^ Here, a lack of calcitonin staining would push the differential away from MTC, just as a lack of thyroglobulin staining would make follicular thyroid disease less likely.

Immunostaining of PTH in cells is possible but difficult because only a small amount of PTH is stored in individual chief cells.^35^, ^36^ However, finding increased PTH levels in the *rinse aspirate* obtained via FNA can provide evidence of a parathyroid adenoma,^37^ with reported sensitivities ranging from 82% to 94%.^11^, ^38^, ^39^ This can be particularly useful in differentiating thyroid nodules from IPAs, which can appear similar on ultrasound.^19^

However, FNA has been linked to a high frequency of fibrosis in the parathyroid gland.^40^ This can make operative localization more difficult. It is common for patients with IPA to have undergone previous unsuccessful neck surgery,^20^, ^22^ making fibrotic tissue more likely and compounding the issue. Reactive changes can also make the gland adherent to surrounding structures, including the recurrent laryngeal nerve (RLN), complicating dissection.^40^ Seven percent of IPAs have been noted to be adjacent to the RLN.^20^ Fibrotic changes also create a histological picture that overlaps with malignancy, making differentiation more difficult microscopically.

#### 3.3.3 Nuclear Medicine imaging techniques

^99m^Technetium‐based scintigraphy (MIBI) has a well‐established role in localizing parathyroid adenomas, and common variants include planar, SPECT and SPECT/CT. Seven studies reported using these techniques, with 1 specifying use of SPECT/CT and 1 specifying use of SPECT (Table 2).

Studies with sample sizes of >10 IPAs have shown that MIBI‐based techniques have a sensitivity of 60%–83% in detecting IPAs.^7^, ^10^, ^12^, ^13^, ^18^ False positives can be due to benign thyroid nodules and malignant thyroid disease,^41^ which can also retain radiotracer. False negatives can be due to smaller adenomas and multiglandular disease and correlate with normal serum Ca^2+^ levels.^42^ Positive sestamibi uptake in the thyroid in a patient with biochemical and clinical evidence of primary hyperparathyroidism should raise suspicion for an IPA. However, due to varying accuracies, a negative scan in such a patient should not be a reason to exclude this diagnosis, and further investigation is advised. The use of multiple localization techniques (e.g., US + sestamibi; US + sestamibi + CT) has been shown to result in a higher accuracy in detecting IPAs than using each technique in isolation.^12^, ^13^

Newer nuclear medicine imaging modalities, including ^11^C‐methionine PET/CT, have been used in localizing parathyroid adenomas.^43^, ^44^ ^11^C‐methionine is a critical amino acid in the composition of pre‐pro‐PTH, the precursor for parathyroid hormone. Consequently, it has been linked to increased specificity for the parathyroid gland.^45^ While our search did not identify any studies that used this technique to localize IPAs, Met PET/CT imaging can accurately identify other ectopic adenomas in those with negative sestamibi imaging.^43^, ^44^ Other radioisotopes are also being introduced. ^18^F‐fluorocholine has the benefit of a longer half‐life compared to ^11^C‐methionine.^46^ It also has a more amenable positron range, which gives it a higher spatial resolution than ^99m^Tc MIBI and ^11^C‐methionine based PET/CT imaging. This allows the detection of smaller adenomas, including intrathyroidal adenomas.^47^, ^48^

Nuclear medicine techniques can also be used intraoperatively to localize IPAs. Following preoperative injection with ^99m^Tc‐Sestamibi, a gamma probe can be used during surgery to scan the thyroid for areas of increased radionuclide uptake. Experience is required with this technique to navigate background radionucleotide counts and avoid false positives, but some centers have reported high localization rates for ectopic adenomas, including IPAs, using this technique.^10^, ^13^

#### 3.3.4 Computed tomography

Five studies described the use of computed tomography (CT) in localizing IPAs (Table 2). In three studies with a sample size <5 IPAs, CT was unable to correctly localize IPAs in any case.^10^, ^12^, ^22^ However, they did not provide information on the underlying imaging features for these cases. Therefore, it is unclear if CT imaging was completely negative, or if a signal was present but interpreted as another pathology (e.g., thyroid nodule). On noncontrast CT imaging, parathyroid adenomas classically appear as distinct hypoattenuating nodules relative to the surrounding thyroid, and some IPAs do appear in this manner.^17^ However, this is not a unique feature as thyroid nodules can also be hypoattenuating and can be mistaken for an IPA.^49^ In conditions such as Hashimoto's thyroid disease, lower iodine levels in the gland can decrease the attenuation of the thyroid, making it more challenging to differentiate parathyroid adenomas.^50^

Two studies reported that around 65%^13^, ^17^ of IPAs were detected successfully using CT only. However, they also had small sample sizes of <10 and did not provide details on the underlying imaging features in successful cases. Using other techniques in combination with CT, such as ultrasound and/or sestamibi, as is typical in real‐world practice, can have higher success rates compared to CT alone.^13^

No studies reported using 4D‐CT in detecting IPAs. However, 4D‐CT can detect smaller parathyroid lesions relative to MIBI and accurately identify ectopic parathyroid adenomas missed by scintigraphy and ultrasound.^51^, ^52^ IPAs tend to be smaller compared to normal parathyroid adenomas.^10^ Differentials to IPAs in the thyroid can exhibit unique features on 4D‐CT, particularly with time‐dependent contrast imaging, which can be helpful in differentiation (Table 3).

**TABLE 3:** Imaging features of IPAs compared to common differentials

	Ultrasound	Sestamibi	CT	MRI
Intrathyroidal parathyroid adenoma	Hypoechoic solid nodule^17^, ^18^, ^19^ Polar vessel supplying adenoma^17^, ^18^, ^19^ Hyperechoic line on the ventral surface^12^, ^17^	Positive or negative uptake^7^, ^10^	Parathyroid adenomas are usually hypoattenuating relative to the thyroid on noncontrast imaging^68^ Homogenous appearance^68^ Usually, greater attenuation in the arterial phase and lower attenuation in the delayed phase relative to the thyroid^68^	Parathyroid adenomas usually have a higher signal on postcontrast T1‐weighted sequences and T2‐weighted imaging^54^
Benign thyroid nodule	Hyperechoic^69^ Regular and well‐defined margins^69^ Coarse or curvilinear calcifications may be present^69^ “Comet tail” sign indicating colloid^69^ Absence of blood flow or perinodular flow only^69^	Usually negative but can have positive uptake^70^	Can be isoechoic or hyperechoic relative to the background thyroid^68^ Can have similar contrast enhancement characteristics to IPAs^68^	Higher signal intensity ratio (SIR) on T2 imaging, and higher apparent diffusion coefficient (ADC) for benign nodules (relative to malignant nodules)^71^ Regular shape and well‐defined margins^71^
Thyroid malignancy	Hypoechoic^69^ Irregular borders^69^ Punctate microcalcifications^69^ Absence of halo around the nodule^69^ Disorganized blood flow on Doppler with intranodular vascularity^69^	Usually negative but can be positive^72^	Can have reduced attenuation relative to benign thyroid nodules without contrast^73^ Irregular shape and calcifications may be present^74^ Evidence of extra‐thyroidal spread^74^	Lower signal intensity ratio (SIR) on T2 imaging, and lower apparent diffusion coefficient (ADC) for malignant nodules (relative to benign nodules)^71^ Irregular shape, ill‐defined margins^71^
Parathyroid cancer	Heterogenous mass^75^ Lobulated contours^75^ Depth‐width ratio >0.5^75^ Disorganized color flow on Doppler^75^	Can display increased signal in delayed images relative to the background^76^	Heterogenous mass^68^ Can have calcifications^68^ Can analyze surrounding tissue for evidence of metastatic spread^77^	Little data on ADC, SIR values Can analyze surrounding tissue for evidence of metastatic spread^77^
Benign or reactive lymph node	Short axis to long axis ratio of <0.5^78^ Echogenic hilum and hypoechoic cortex^78^ Hilar vascularity only or avascular^78^	Usually, negative^79^	Hypoattenuating on noncontrast imaging^68^ Greater attenuation in the delayed phase, in contrast to parathyroid adenomas^68^	Higher apparent diffusion coefficient (ADC) than malignant nodes^80^ Regular margins around a homogenous mass^80^
Metastatic lymph node	Short axis to long axis ratio of >0.5^78^ Sharp borders^78^ Eccentric hypertrophy of the cortex due to tumor infiltration^78^ Hypoechoic or hyperechoic^78^ Calcifications can be present^78^ Peripheral vascularity or mixed peripheral and hilar vascularity^78^	Can be positive, but studies with metastatic axillary lymph nodes showed low sensitivity with MIBI^81^	Can have a heterogeneous appearance due to tumor infiltration, necrosis, and calcification^82^ Poorly differentiated margins and capsular enhancement can indicate extracapsular spread^83^	Lower apparent diffusion coefficient (ADC) than benign nodes^80^ More likely to have little/no hilar fat compared to benign nodes^84^ Irregular margins around heterogeneous mass^80^

Abbreviations: ADC, apparent diffusion coefficient; CT, computed tomography; MRI, magnetic resonance imaging; SIR, signal intensity ratio.

As with ultrasound, the presence of a polar feeding vessel can help with localization. This feature has been reported in 60% of eutopic parathyroid adenomas on 4D‐CT,^53^ differing from the hilar blood supply associated with lymph nodes.

#### 3.3.5 Magnetic resonance imaging

Two studies reported varying sensitivities of magnetic resonance imaging (MRI) in detecting IPAs, ranging from 25% to 80%.^10^, ^22^ In these studies, the sample sizes for MRI were small (<10) and unique imaging features of IPAs were not reported. Eutopic adenomas often display low‐intensity signals on T1‐weighted sequences, with enhancement following contrast medium administration and a high signal on T2 weighted sequences.^54^ In those with iodine contrast allergies, it may offer a substitute for CT scanning.

#### 3.3.6 Selective venous sampling

Selective venous sampling (SVS) is an invasive localization technique where blood is sampled from veins draining the parathyroid gland. Sample sites include the internal jugular, brachiocephalic, and the superior, middle, and inferior thyroid veins. Elevated PTH values at particular sample sites can be used to localize the IPA with knowledge of drainage patterns.^22^ Due to its invasive nature, this technique is rarely used and is often only considered after inconclusive non‐invasive imaging tests. Our search identified two studies that reported the use of SVS in detecting IPAs. Both had a sample size of ≤3 with this technique and sensitivities varied from 33% to 100% in locating IPAs.^22^, ^23^

While this technique can allow a precise determination of the site of elevated PTH levels, it is unable to differentiate between parathyroid hyperplasia and parathyroid adenoma. Other factors that prevent routine use include the length and cost of the procedure.

#### 3.3.7 Emerging techniques

Parathyroid glands display autofluorescence in the infrared spectrum that can be picked up by a near‐infrared (NIR) camera or spectroscopy. When excited at a wavelength of around 800 nm, parathyroid glands spontaneously emit light in the 820–830 nm range.^55^ This signal usually greatly exceeds the fluorescence from surrounding tissue such as the thyroid.^56^ While there have been individual case reports of using autofluorescence to successfully localize IPAs,^57^ we did not identify studies reporting sensitivities and specificities for detecting this variant. On NIR imaging, colloid nodules can have a high‐intensity signal and can be mistaken for an IPA.^58^ Moreover, some complete IPAs can be deeply enveloped by the thyroid and might be missed using this technique. Other techniques trialed in detecting parathyroid glands include optical coherence tomography (OCT) and the use of staining dyes such as methylene blue and 5‐aminolevulinic acid (5‐ALA).^59^, ^60^ However, operational complexities associated with OCT, and reports of toxicity with staining agents have limited their widespread use. We did not identify any studies using these techniques to localize IPAs.

#### 3.3.8 Imaging features of important differentials

Several structures can mimic IPAs on imaging. Thyroid nodules frequently co‐exist with IPAs^19^ and several reports have highlighted concurrent IPA and nonmedullary thyroid carcinoma.^61^, ^62^, ^63^ Moreover, there have also been several reports of intrathyroidal parathyroid carcinoma.^64^, ^65^, ^66^, ^67^ Other possible differentials include benign and metastatic lymph nodes, where they might be mistaken for partial IPAs if they are adjoining the thyroid. For reference, the differing features of IPAs and potential differentials using common imaging techniques are highlighted in Table 3.^68^, ^69^, ^70^, ^71^, ^72^, ^73^, ^74^, ^75^, ^76^, ^77^, ^78^, ^79^, ^80^, ^81^, ^82^, ^83^, ^84^

### 3.4 Surgical considerations

Eight studies provided information on surgical techniques (lobectomy, thyroidotomy, and enucleation) and/or intraoperative location of IPAs (Table 2).

#### 3.4.1 Lobectomy

Complete lobectomy involves removing one thyroid lobe, while partial lobectomy involves removing only part of one lobe. Both techniques have been reported to excise IPAs^16^, ^20^ with preference often dictated by how confidently the surgeon can locate the IPA during surgery. This is often challenging—fibrotic tissue from previous surgical attempts to locate the adenoma can make the operative field difficult to navigate. During the operation, the thyroid can be palpated to locate a potential IPA^16^ guided by localization techniques. If this reveals a distinct lesion, partial lobectomy is sometimes attempted. If a lesion cannot be palpated, partial or complete lobectomy may still be indicated, especially if imaging/FNA suggests an IPA, and there is no evidence of a eutopic adenoma or an ectopic adenoma at other locations. In either case, a thorough search for all four parathyroid glands before thyroid lobectomy, irrespective of negative findings from prior surgical attempts, can reduce failure rates. Goodman et al.^20^ studied a large cohort of 1000 patients with persistent primary hyperparathyroidism following a first unsuccessful surgery. At the end of the first surgery, approximately 75% had a thyroid lobectomy as a last resort to remove a potential IPA. Lobectomy was performed after failed attempts to localize a normal adenoma. At reoperation, a normal adenoma (i.e., not intrathyroidal) was found in the neck >99% of the time, highlighting the need for careful dissection and searching. If the latter is performed comprehensively, including a thorough search at other common ectopic sites such as the retropharyngeal and trachea‐esophageal areas, lobectomy can have high success rates.^15^

As ascertained from a systematic review, the risk of clinical hypothyroidism after thyroid lobectomy is 4%.^85^ As a result, an appropriate level of suspicion for an IPA is indicated before proceeding with lobectomy (e.g., after thorough search for a eutopic adenoma is negative^15^, ^21^ and/or if the lesion has sufficient features of IPA on imaging).

#### 3.4.2 Intraoperative location of IPAs

In a different cohort of 10 000 patients undergoing first‐time parathyroidectomy, Goodman et al. encountered 72 IPAs and found that most (90%) were located in the lower lateral quadrant of the thyroid, with 7% in the lateral thyroid border adjacent to the recurrent laryngeal nerve and only 3% in the superior pole.^20^ Moreover, the majority of IPAs (98%) were inferior parathyroid glands, confirmed by locating the superior parathyroid on the ipsilateral side; this finding has also been reported by several others.^1^, ^3^, ^10^, ^86^

However, the authors only counted complete IPAs and did not include partial IPAs when reporting localization. Partial IPAs could potentially be distributed more frequently in other areas.

#### 3.4.3 Other excision techniques

Some centers have reported high success rates using more targeted excision such as IPA enucleation or thyroidotomy, instead of lobectomy.^10^, ^13^ Targeted enucleation relies on a more precise determination of location intraoperatively, and these centers have used radio‐guided techniques, including gamma probes, to facilitate this. Other advantages include a lower risk of bleeding intraoperatively and postoperatively compared to traditional lobectomy. However, the use of gamma probes to aid localization during surgery is operator‐dependent and requires experience.

Intraoperative PTH monitoring can also be used to evidence IPA removal; some studies report that a high number of IPAs (32%) were found on further exploration following inadequate PTH drops after an excision.^10^

Minimally invasive parathyroidectomy is increasingly used when imaging provides strong localizing evidence of an adenoma. However, this is rarely the case for IPAs and leaves little room for adequate surgical exploration.

### 3.5 Histopathology

Two studies provided information on the size and weight of IPAs on resection, or on their macroscopic and microscopic appearances. Macroscopically, IPAs can be confirmed as complete or partial intrathyroid adenomas, depending on the extent of thyroid envelopment. IPAs also tend to be smaller than eutopic adenomas. In a cohort of 53 IPAs, Mazeh et al.^10^ reported that IPAs had a mean weight of 325 mg, almost two times lower than the mean weight for non‐intrathyroidal adenomas (772 mg). Macroscopically, IPAs can have a well‐circumscribed, nodular, or lobulated contour.^9^ In a cohort of 10 IPAs, Chandramohan et al.^9^ reported that most had a firm texture, had a tan/yellow appearance, and were covered by a thin capsule.

Microscopically, they can show features associated with parathyroid adenomas, including regular arrangements of chief cells punctuated by fine capillary networks.^9^ However, they can also have high proportions of oxyphil, transitional, or water‐clear cells,^87^, ^88^ although the prevalence of this is unknown. Thyroid pathology, including malignancy and nodular disease, is often seen in the removed thyroid segment.^9^

## 4 DISCUSSION

The aim of this review is to map the current literature on intrathyroid parathyroid adenomas, focusing on areas of clinical importance (e.g., imaging, surgical considerations) and highlighting gaps for further research. Due to the varied nature of the categories of information collected (Table 1), a scoping review was deemed best suited for this approach.

### 4.1 Clinical presentation

Studies that reported clinical symptoms for IPAs tended to focus on the type of symptom without commenting on the severity of each symptom. Due to delays in diagnosing ectopic parathyroid adenomas, patients with these variants can present with progressive symptoms of hypercalcemia, such as severe bone disease.^4^ Future studies should consider noting the extent of PHPT features in those with IPAs (e.g., recording the frequency of kidney stones, as opposed to the presence of this feature alone).

### 4.2 Localization techniques

Current imaging modalities available to localize IPAs range from ultrasound to selective venous sampling. This review also introduces emerging techniques including autofluorescence. Imaging features of IPAs, particularly on ultrasound, along with common differentials, are highlighted in Table 3.

Despite a systematic literature search, data on imaging features of IPAs, especially using modalities such as 4D‐CT, ^11^C‐Met PET/CT, SVS, and MRI are sparse. Further studies using larger sample sizes will be required to determine unique features and to discern sensitivities and specificities. This will be useful in informing the order of imaging tests to accurately localize IPAs. Due to the rarity of this variant, multicenter studies combining IPA cohorts might be required to achieve this goal.

Many studies using MRI and CT report very low accuracies.^10^, ^12^ However, this is difficult to interpret due to small sample sizes. Additionally, these modalities are often reserved for difficult cases where first‐line imaging with ultrasound +/− sestamibi is inconclusive, introducing a selection bias. In practice, imaging results from CT/ MRI are often interpreted in conjunction with results from techniques such as ultrasound. When used in combination, as is often the case in real‐world practice, they have a higher accuracy in detecting IPAs.^13^

Due to small sample sizes that make it difficult to interpret reported sensitivities and specificities, future studies should also consider reporting imaging *features* of IPAs and comparing it to co‐existing pathology (e.g., thyroid nodules).

### 4.3 Surgical considerations and histopathological features

Techniques for removing IPAs include thyroid lobectomy (partial or full), thyroidotomy and enucleation. For lobectomy, higher success rates have been reported if it is performed after a comprehensive search for normal adenomas and other ectopic adenomas.^20^ Common ectopic sites include the thymus, trachea‐esophageal groove, retropharyngeal space, and superior mediastinum.^3^, ^7^ Intraoperatively, evidence suggests most IPAs correspond to the inferior parathyroid gland in the lower thyroid lobe.^1^, ^3^, ^10^, ^13^, ^20^ Less common but other possible locations include the superior lobe and the dorsal surface of thyroid lobe near the recurrent laryngeal nerve.

While there were a number of retrospective studies that described a center's experience with a particular surgical technique (i.e., lobectomy), there were no studies that randomized IPAs into different surgical methods for removal. The optimum technique likely depends on a variety of patient, surgeon, and center‐dependent factors. For instance, a lack of fibrotic tissue could make an IPA easier to locate intraoperatively and might allow partial lobectomy. Alternatively, the availability of intraoperative localization techniques such as gamma probes, and experience using them, might facilitate enucleation.

An IPA can be “complete” when it is fully enveloped by thyroid tissue, or “partial” when more than 50% of its surface, but not the whole surface, is covered by thyroid tissue. This categorization is important because the accuracy of localization techniques such as ultrasound varies with IPA type.^19^ In this review, some studies only included complete IPAs, while others included both complete and partial IPAs, but analyzed them together (Table 2). Others did not specify the type of IPA included. This makes it difficult to compare and interpret reported localization accuracies between different studies.

IPA type could also theoretically impact the success rates of different surgical techniques. As partial IPAs are not completely enveloped, they might be easier to visualize during surgery and allow more targeted removal (e.g., partial lobectomy or enucleation). Therefore, a study with a high proportion of partial IPAs could report a high success rate with these targeted techniques. However, these findings might be less generalizable in a cohort with a large number of complete IPAs, which can be well‐hidden in the thyroid. For these reasons, future studies should clearly specify which type of IPAs they are including, and should consider stratifying their IPA population into these two groups and analyzing them separately.

## 5 CONCLUSION

Our scoping review maps the current literature on IPAs, focusing on clinical presentation, localization techniques, and intraoperative factors. Imaging from one modality is rarely conclusive on its own; overall impression from a range of tests is often performed to guide management. However, little data is available on imaging techniques such as CT and MRI and is an area for further research. While an IPA can be present throughout the thyroid, the most common location appears to be in the inferior thyroid lobe, with most IPAs corresponding to the inferior parathyroid gland. A thorough search for normal adenomas and at other ectopic sites before thyroid lobectomy is associated with higher success rates. Further research should consider stratifying IPAs into complete and partial subtypes and aim to compare the success rates of different surgical techniques such as lobectomy and enucleation.

## CONFLICT OF INTEREST

The authors declare that there is no conflict of interest that could be perceived as prejudicing the impartiality of the research reported.

## Supporting information


**Appendix S1.** Supporting Information.

## Data Availability

This is not applicable as no new data were created or analyzed in this scoping review.
