# Supplementary material for: Intrathyroidal parathyroid adenomas: Scoping review on clinical presentation, preoperative localization, and surgical treatment
Source: Head Neck. 2022 Dec 23;45(3):706–20. doi: 10.1002/hed.27287 (PMC10108101; doi:10.1002/hed.27287)
Supplement: Supplementary file 1 — Appendix S1. Supporting Information. [file HED-45-706-s001.docx]

Supplementary file 1

Search strategy for the scoping review

**Database:**
Ovid MEDLINE(R) and Epub Ahead of Print, In-Process, In-Data-Review & Other Non-Indexed Citations, Daily and Versions <1946 to May 30, 2022>

| **#** | **Query** | **Results from 30 May 2022** |
| --- | --- | --- |
| 1 | Intrathyroid* parathyroid adenoma*.mp. | 75 |
| 2 | (intrathyroid* or intra-thyroid*).mp. | 1,524 |
| 3 | (parathyroid* adj3 (neoplasm* or cancer* or adenoma*)).mp. | 10,334 |
| 4 | exp parathyroid neoplasms/ | 8,302 |
| 5 | exp hyperparathyroidism, primary/ | 3,535 |
| 6 | (hyperparathyroid* adj primary).mp. | 3,580 |
| 7 | 3 or 4 or 5 or 6 | 12,581 |
| 8 | 2 and 7 | 198 |
| 9 | 1 or 8 | 198 |

**Database:**
Embase <1974 to 2022 May 30>

| **#** | **Query** | **Results from 30 May 2022** |
| --- | --- | --- |
| 1 | Intrathyroid* parathyroid adenoma*.ti,ab. | 107 |
| 2 | (intrathyroid* or intra-thyroid*).ti,ab. | 1,830 |
| 3 | (parathyroid* adj3 (neoplasm* or cancer* or adenoma*)).ti,ab. | 7,590 |
| 4 | exp parathyroid adenoma/ | 7,258 |
| 5 | exp primary hyperparathyroidism/ | 10,174 |
| 6 | (hyperparathyroid* adj primary).ti,ab. | 47 |
| 7 | 3 or 4 or 5 or 6 | 16,301 |
| 8 | 2 and 7 | 258 |
| 9 | 1 or 8 | 258 |

**Database:**

Cochrane Library May 30 2022

ID Search Hits

#1 (Intrathyroid* parathyroid adenoma):ti,ab,kw 1

#2 (intrathyroid* or intra-thyroid*):ti,ab,kw 37

#3 (parathyroid neoplasm):ti,ab,kw 58

#4 (parathyroid cancer):ti,ab,kw 166

#5 (parathyroid adenoma):ti,ab,kw 77

#6 MeSH descriptor: [Parathyroid Neoplasms] explode all trees 40

#7 MeSH descriptor: [Hyperparathyroidism, Primary] explode all trees 77

#8 (primary hyperparathyroid*):ti,ab,kw 587

#9 #3 OR #4 OR #5 OR #6 OR #7 OR #8 801

#10 #2 AND #9 1

#11 #1 OR #10 1
